# Development of a scale for early prediction of refractory *Mycoplasma pneumoniae* pneumonia in hospitalized children

**DOI:** 10.1038/s41598-021-86086-5

**Published:** 2021-03-23

**Authors:** Ying Bi, Yifan Zhu, Xiao Ma, Jiejing Xu, Yun Guo, Tianyu Huang, Siqing Zhang, Xin Wang, Deyu Zhao, Feng Liu

**Affiliations:** 1 Department of Respiratory Medicine, Children’s Hospital of Nanjing Medical University, 72 Guangzhou Road, Nanjing, China; 2 Xuzhou Children’s Hospital, Xuzhou Medical University, Xuzhou, China; 3 Department of Pediatrics, The Second People’s Hospital of Changzhou, Affiliate Hospital of Nanjing Medical University, Changzhou, Jiangsu China; 4 Department of Respiratory Medicine, The Affiliated Wuxi Children’s Hospital of Nanjing Medical University, Wuxi, China

**Keywords:** Pathogens, Paediatric research, Respiratory tract diseases

## Abstract

Now there is no clinical scale for early prediction of refractory *Mycoplasma pneumoniae* pneumonia (RMPP). The aim of this study is to identify indicators and develop an early predictive scale for RMPP in hospitalized children. First we conducted a retrospective cohort study of children with *M. pneumoniae* pneumonia admitted to Children’s Hospital of Nanjing Medical University, China in 2016. Children were divided into two groups, according to whether their pneumonia were refractory and the results were used to develop an early predictive scale. Second we conducted a prospective study to validate the predictive scale for RMPP in children in 2018. 618 children were included in the retrospective study, of which 73 with RMPP. Six prognostic indicators were identified and included in the prognostic assessment scale. The sensitivity of the prognostic assessment scale was 74.0% (54/73), and the specificity was 88.3% (481/545) in the retrospective study. 944 children were included in the prospective cohort, including 92 with RMPP, the sensitivity of the prognostic assessment scale was 78.3% (72/92) and the specificity was 86.2% (734/852). The prognostic assessment scale for RMPP has high diagnostic accuracy and is suitable for use in standard clinical practice.

## Introduction

*Mycoplasma pneumoniae* is one of the important pathogens that cause childhood community acquired pneumonia. The incidence of *M. pneumoniae* infection does not differ by sex, but it varies substantially by age. It is most common in preschool and school age children. The infection rate of pneumonia in children over 5 years old can be as high as 50%^1,2^. Pneumonia caused by *M. pneumoniae* infection is generally self-limiting, but sometimes is refractory. After regular treatment, lung lesions can still recur or be prolonged, resulting in residual structural and/or functional lung damage, often manifested as mosaic signs and bronchiectasis^3^. These sequelae often cause repeated lung infections in children, and have a significant impact on the lung function of adults, which is also closely related to the occurrence of asthma^4–6^. With the incidence of refractory *M. pneumoniae* pneumonia in children steadily increasing and some case fatalities, early diagnosis and treatment of refractory *M. pneumoniae* pneumonia is particularly important^7^.

For the prognosis of adult community acquired pneumonia, A variety of predictive indicators such as the Pneumonia Severity Index and CURB-65 score have been developed to determine the prognosis of community-acquired pneumonia in adults^8,9^. However, given the practicality of these scales and age limitations, they cannot be directly applied to children.

There has been some research on the predictors of refractory *M. pneumoniae* pneumonia. Large-scale pulmonary morphogenesis, extrapulmonary complications, and elevated CRP and LDH are clinically relevant risk factors for refractory *M. pneumoniae* pneumonia^10–12^. However, the current prediction methods often use only a single indicator to judge the prognosis, or there are few clinical data and no prospective verification. The indicators included in some studies are not readily available clinically, and in some studies, the outcome was complications caused by refractory *M. pneumoniae* pneumonia, rather than predictors of refractory *M. pneumoniae* pneumonia^13,14^. Therefore, the aim of this study was to use multiple simple indicators to develop a scale for early prediction of refractory *M. pneumoniae* pneumonia in hospitalized children.

## Methods

### Ethics

The study was approved by the institutional ethics committee of Children’s Hospital Affiliated to Nanjing Medical University (Approval number: 201801126-1), and was registered in the Chinese Clinical Trial Registry (Registration number: ChiCTR1800015673). All methods were performed in accordance with the Declaration of Helsinki.

### Informed consent

Informed consent was obtained from all subjects or, if subjects are under 18, from a parent and/or legal guardian.

### Patients and groups

**Figure 1:**
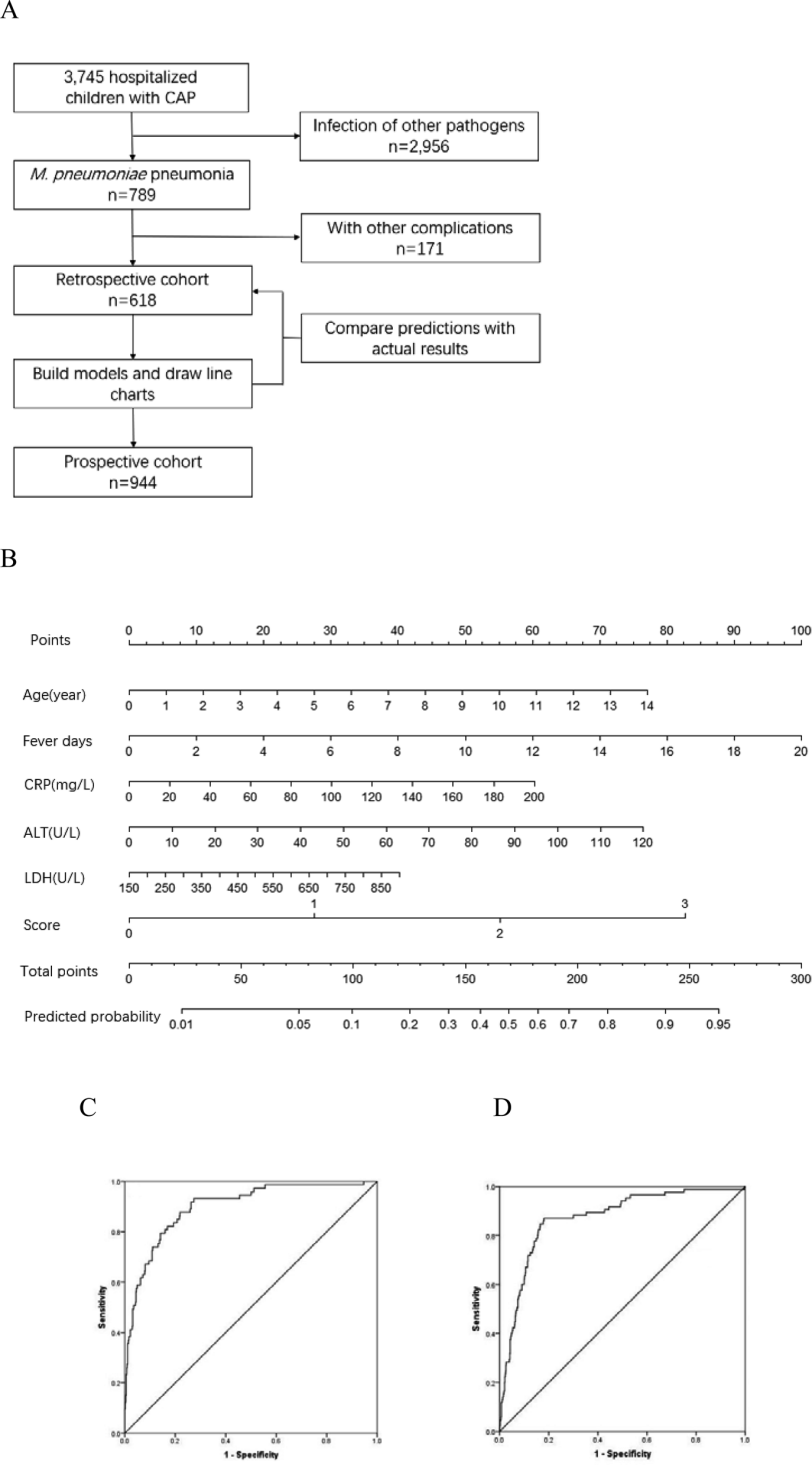
(**A**) Study flow. CAP, community-acquired pneumonia; RMPP, refractory *M. pneumoniae* pneumonia. (**B**) The first line is the score corresponding to each indicator value. The following is the index included in the scale, and finally the calculated total score and predicted probability. When using, the table should be scaled up and printed on paper, and the score should be calculated using a tool such as a ruler. (**C/D**) Scale for predicting refractory *M. pneumoniae* pneumonia by receiver operator characteristic curves. (**C**) In the retrospective cohort; (**D**) In the prospective cohort.

A flowchart of our research is provided in Fig. 1A. We conducted a retrospective cohort study among children admitted to the Children's Hospital of Nanjing Medical University with *M. pneumoniae* pneumonia from January to December 2016. This was followed by a prospective cohort from January to December 2018. All children were first seen in Children’s Hospital. *M. pneumoniae* infection was confirmed by polymerase chain reaction testing of nasopharyngeal swab specimens.

Patients with immune deficiencies, chronic diseases, heart diseases or who were using immunosuppressive drugs were excluded. All those enrolled had negative tuberculosis IgM or purified protein derivative tests. In addition, their nasopharyngeal secretions were negative for respiratory syncytial viruses, influenza viruses, adenovirus, parainfluenza virus, and *Chlamydia trachomatis*. The subjects also had negative bacterial cultures of nasopharyngeal secretions and double-negative blood cultures. Consent for participation was obtained.

Refractory *M. pneumoniae* pneumonia is defined as a case with prolonged fever accompanied by deterioration of radiological findings despite appropriate management and treatment with a macrolide antibiotic for ≥ 7 days^15^. On the basis of this definition, we reviewed patients’ medical records and divided them into 2 groups: RMPP group and non-RMPP group.

### Data collection and study variables

We collected data on demographic and clinical characteristics including age, sex, fever days on admission, and chest imaging findings; and laboratory test results including complete blood count, C-reactive protein, alanine aminotransferase, aspartate aminotransferase, lactate dehydrogenase and creatine kinase. After preliminary screening of all indicators, statistically significant indicators were selected for regression analysis.

### Respiratory pathogens

Nasopharyngeal aspirates were tested for respiratory pathogens using a real-time, multiplex polymerase chain reaction assay in our hospital’s clinical virology laboratory. The specific pathogens identified included influenza A and B, respiratory syncytial viruses, adenovirus, parainfluenza virus, *C. trachomatis*, and *M. pneumoniae*. A positive polymerase chain reaction result for *M. pneumoniae* was a copy number of > 2,500/mL(ACON Biotech Co.,Ltd, Hangzhou, China). Bacterial culture results based on nasopharyngeal aspirates and blood were obtained from the hospital’s microbiology laboratory^16^.

### Statistical analysis

Statistical analysis was performed with SPSS Version 20.0 (IBM Corp, Armonk, NY, USA) and R Version 3.5.3 (R Foundation for Statistical Computing, Vienna, Austria), and *P* < 0.05 was considered statistically significant. Categorical variables were analyzed using the chi-square test. Normally distributed continuous data were analyzed using t tests, and non-normally distributed measurement data were analyzed using Mann–Whitney U tests.

Multivariate analysis was performed using a stepwise logistic regression model. R software was used to transform the final regression model into a nomogram. Receiver operating characteristic (ROC) curves were used to analyze the regression model for prediction of refractory *M. pneumoniae* pneumonia. Calculate the sensitivity and specificity of the predictive scale.

## Results

### Patient characteristics and laboratory findings

The clinical characteristics of the two cohort patients are shown in Table 1.We enrolled 618 patients in retrospective cohort. There were 73 patients in the RMPP group, and 545 patients in the non-RMPP group. The characteristics of the patients in the retrospective cohort on admission are summarized in Table 2.

**Table 1:** Admission characteristics of the children in the retrospective and prospective cohorts.

	Retrospective cohort (n = 618)	Prospective cohort (n = 944)
Age (years)	3.84 ± 2.66	4.47 ± 2.70
Sex: male	338 (54.7%)	516 (54.7%)
Fever days	4.78 ± 3.41	5.09 ± 3.76
WBC (× 10^9^/L)	9.45 ± 4.23	9.75 ± 4.46
Neutrophils (%)	55.08 ± 16.52	55.71 ± 17.47
Neutrophils (× 10^9^/L)	5.37 ± 3.32	5.52 ± 3.51
CRP (mg/L)	16.79 ± 20.22	15.93 ± 20.19
HB (g/L)	122.90 ± 10.46	125.19 ± 14.80
PLT (× 10^9^/L)	276.48 ± 105.23	294.10 ± 118.04
AST (U/L)	34.82 ± 20.48	30.59 ± 24.38
ALT (U/L)	22.18 ± 31.05	21.09 ± 27.82
LDH (U/L)	391.02 ± 225.65	359.15 ± 138.51
CK (U/L)	100.56 ± 119.99	98.57 ± 109.73
**Pleural effusion**		
Small	5.34% (33/618)	5.61% (53/944)
Medium to large	8.25% (51/618)	7.84% (74/944)
Atelectasis or large area of lung consolidation	10.36% (64/618)	11.65% (110/944)

Values are presented as mean ± SD.

*ALT* alanine aminotransferase, *AST* aspartate aminotransferase, *CK* creatine kinase, *CRP* C-reactive protein, *HB* hemoglobin, *LDH* lactate dehydrogenase, *PLT* platelets, *WBC* white blood cells.

**Table 2:** Admission characteristics of children with *Mycoplasma pneumoniae* pneumonia in the retrospective cohort according to their subsequent clinical outcome.

Characteristic	Non-RMPP (n = 545)	RMPP (n = 73)	*P* Value
Age	3.61 ± 2.57	5.55 ± 2.71	< 0.001
Sex: male	300 (55.0%)	38 (52.1%)	0.630
Fever days	4.39 ± 3.15	7.66 ± 3.86	< 0.001
WBC (× 10^9^/L)	9.40 ± 4.21	9.82 ± 4.39	0.414
Neutrophil (%)	53.94 ± 16.22	63.63 ± 16.36	< 0.001
Neutrophils (× 10^9^/L)	5.22 ± 3.25	6.45 ± 3.70	0.008
CRP (mg/L)	15.08 ± 16.64	29.66 ± 34.93	0.001
HB (g/L)	122.78 ± 10.37	123.82 ± 11.17	0.424
PLT (× 10^9^/L)	275.56 ± 104.82	283.32 ± 108.80	0.555
AST (U/L)	33.76 ± 17.45	42.04 ± 34.06	0.045
ALT (U/L)	18.76 ± 14.16	45.64 ± 73.89	0.003
LDH (U/L)	379.78 ± 223.97	474.93 ± 221.89	0.001
CK (U/L)	102.96 ± 125.22	83.08 ± 66.66	0.18
**Pleural effusion**			
Small	5.14% (28/545)	6.85% (5/73)	0.575
Medium to large	4.95% (27/545)	32.88% (24/73)	< 0.001
Atelectasis or Large area lung consolidation	5.50% (30/545)	46.58% (34/73)	< 0.001

Values are presented as mean ± SD.

*ALT* alanine aminotransferase, *AST* aspartate aminotransferase, *CK* creatine kinase, *CRP* C-reactive protein, *HB* hemoglobin, *LDH* lactate dehydrogenase, *PLT* platelet, *RMPP* refractory *M. pneumoniae* pneumonia, *WBC* white blood cell.

There was no significant difference in sex distribution between the 2 groups. The average age and fever days were significantly greater in the RMPP group than that in the non-RMPP group. Compared with the non-RMPP group, significantly more patients in the RMPP group had atelectasis or lobar or segmental lung consolidation, and moderate to large pleural effusions than those in the non-RMPP group.

Compared with the non-RMPP group, the RMPP group showed significantly higher levels of C-reactive protein, neutrophil %, neutrophils (absolute value), aspartate aminotransferase, alanine aminotransferase, and lactate dehydrogenase. The other laboratory findings did not differ significantly between the two groups.

### Chest imaging score

In order to be able to incorporate chest imaging findings into regression analysis, we created a new indicator, the chest imaging score (Table 3).

**Table 3:** Chest imaging score.

Chest imaging findings	Yes	No
**Pleural effusion**		
Small	1	0
Medium to large	2	0
Atelectasis or Large area lung consolidation	1	0
Chest imaging score		

A small amount of pleural effusion: the angle of the costal diaphragm becomes dull; a medium amount of effusion: a large uniform dense shadow in the lower pleural cavity, the upper boundary is curved, the concave surface is upward, and the highest point is in the armpit; Even shadow, the mediastinum is pushed to the opposite side; Large-area lung consolidation: occupying a segment of the lung or above the range of the lung lobes (range over 2/3 of the lung lobes), can involve single or multilobe lesions^17–19^.

### Logistic regression and nomogram

All variables that were statistically significant in the comparison between groups were considered for inclusion in the logistic regression analysis. The variables were screened using the maximum likelihood ratio forward stepwise regression method. Finally, age, fever days, C-reactive protein, alanine aminotransferase, lactate dehydrogenase and chest imaging score were included in the predictive model. (Table 4). The final predictive model is shown as a nomogram in Fig. 1B.

**Table 4:** Logistic regression analysis predictors of *M. pneumoniae* pneumonia.

Variable	β	SE	Wald	*P*	Odds ratio	95% CI For OR	
						Lower	Upper
Age	0.157	0.057	7.731	0.005	1.170	1.048	1.307
Fever days	0.151	0.044	11.662	0.001	1.163	1.067	1.269
CRP	0.017	0.007	6.155	0.013	1.017	1.004	1.031
ALT	0.026	0.008	10.472	0.001	1.026	1.010	1.042
LDH	0.002	0.001	5.534	0.019	1.002	1.000	1.004
Chest imaging score			51.874	0.000			
1	2.544	0.365	48.553	0.000	12.733	6.225	26.046
2	1.373	0.523	6.885	0.009	3.948	1.416	11.014
3	2.167	0.632	11.765	0.001	8.731	2.531	30.116

*ALT* alanine aminotransferase, *CRP* C-reactive protein, *LDH* lactate dehydrogenase.

### Prospective cohort

From January to December 2018, 944 children admitted to our hospital with *M. pneumoniae* pneumonia were enrolled in the prospective cohort study. The characteristics of the patients in the prospective cohort are shown in Table 5.

**Table 5:** Admission characteristics of children with *Mycoplasma pneumoniae* pneumonia in the prospective cohort according to their subsequent clinical outcome.

Variable	Non-RMPP (n = 852)	RMPP (n = 92)	*P* value
Age	4.35 ± 2.68	5.63 ± 2.66	< 0.001
Sex: male	468 (384)	48 (44)	0.614
Fever days	4.88 ± 3.72	7.00 ± 3.65	< 0.001
WBC (× 10^9^/L)	9.70 ± 4.46	10.16 ± 4.51	0.352
Neutrophil (%)	55.05 ± 17.45	61.92 ± 16.54	< 0.001
Neutrophils (× 10^9^/L)	5.43 ± 3.45	6.30 ± 4.02	0.048
CRP (mg/L)	14.68 ± 18.44	27.55 ± 29.85	< 0.001
HB (g/L)	125.11 ± 13.37	126.02 ± 24.52	0.575
PLT (× 10^9^/L)	294.23 ± 117.93	292.85 ± 119.66	0.915
AST (U/L)	30.05 ± 21.74	35.64 ± 41.39	0.205
ALT (U/L)	20.03 ± 21.74	30.91 ± 59.10	0.083
LDH (U/L)	355.25 ± 137.18	395.33 ± 146.20	0.013
CK (U/L)	100.81 ± 113.39	77.65 ± 63.16	0.056
**Pleural effusion**			
Small	4.11% (35/852)	19.57% (18/92)	< 0.001
Medium to large	6.22% (53/852)	22.83% (21/92)	< 0.001
Atelectasis or Large area lung consolidation	6.46% (55/852)	59.78% (55/92)	< 0.001

Values are presented as mean ± SD.

*ALT* alanine aminotransferase, *AST* aspartate aminotransferase, *CK* creatine kinase, *CRP* C-reactive protein, *HB* hemoglobin, *LDH* lactate dehydrogenase, *PLT* platelets, *RMPP* refractory *M. pneumoniae* pneumonia, *WBC* white blood cells.

### Receiver-operating characteristic curve analysis

In the retrospective cohort, the area under the curve for the predictive scale was 0.899 (95% CI 0.860–0.937) as determined by ROC curve analysis (Fig. 1C). In the prospective cohort, the area under the curve was 0.871 (95% CI 0.830–0.911, Fig. 1D).

The optimal cutoff of the scale for predicting refractory *M. pneumoniae* pneumonia was 0.2, with a sensitivity of 74.0%, specificity of 88.3%, and consistency rate of 86.6% in the retrospective cohort. The optimal cutoff in the prospective cohort was also 0.2, with a sensitivity of 78.3%, specificity of 86.2%, and consistency rate of 85.4% (Table 6).

**Table 6:** Predictive value of the predictive scale.

Cohort	Area under the curve	Cutoff	Sensitivity (%)	Specificity (%)	Positive LR	Negative LR	Consistency rate (%)
Retrospective	0.899	0.2	74.0	88.3	6.3	0.3	86.6
Prospective	0.871	0.2	78.3	86.2	5.7	0.3	85.4

Receiver operating characteristic curve analysis was performed with suitable parameters to create cutoffs to determine the predicted probability with regard to refractory *M. pneumoniae* pneumonia. LR, likelihood ratio.

## Discussion

Currently, the majority viewpoint is that the main pathogenic mechanism for the lung damage that occurs in some children with *M. pneumoniae* pneumonia is due to inflammatory damage mediated by human autoimmune function^20^. The symptoms of *Mycoplasma pneumoniae* pneumonia have a rapid onset and are changeable. After treatment, *M. pneumoniae* pneumonia can also cause serious complications^21–24^.

In order to early predict refractory *M. pneumoniae* pneumonia and reduce the incidence of complications and long-term lung damage, we identified 6 prognostic indicators, including age, fever days, CRP, ATL, LDH, and chest imaging findings. The incidence of refractory *M. pneumoniae* pneumonia in the retrospective cohort increased with age, suggesting that the pathogenic mechanism in refractory *M. pneumoniae* infection is related to an excessive immune response^25^. A persistent fever and CRP are common clinical indicators of infection. LDH is also considered to replace inflammatory cytokines such as IL-18 as useful indicators for predicting refractory *M. pneumoniae* pneumonia^26^.These indicators were higher in those in the RMPP group than in those in the non-RMPP group, indicating that the children with refractory *M. pneumoniae* pneumonia have a more pronounced inflammatory responses. Hepatic dysfunction is a common extrapulmonary injury after *M. pneumoniae* infection. Both AST and ALT can reflect hepatocyte function, but ALT is often considered to be a specific indicator of liver injury in patients with *M. pneumoniae* pneumonia^27^.

There are many factors affecting the prognosis of children with pneumonia, but because there is no support for big data, there are no established criteria for predicting which children are at risk of a poor outcome. Some existing prediction scales often lack the universality of clinical application because of a bias of the original data, or are derived from the improved adult scale and has a narrower scope of application^28–30^. Some previous reports have also shown that increasing age, severe chest imaging findings, and elevated inflammatory markers can effectively predict the occurrence of refractory *M. pneumoniae* pneumonia and its complications. Clinical features combined with laboratory results can improve the diagnosis of refractory *M. pneumoniae* pneumonia^31^.

The predictive power of the scale obtained in this study on refractory *M. pneumoniae* pneumonia has good performance in both retrospective and prospective cohorts. The area under the ROC curve in the retrospective and prospective cohort was 0.899 and 0.875, respectively, indicating that the predictive scale can correctly distinguish between children with refractory *M. pneumoniae* pneumonia and those with simple disease. The scale has high sensitivity and specificity in the two cohorts. Compared with other studies, the clinical indicators included in this study are relatively simple and easy to obtain, which is more conducive to application in clinical work.

## Conclusions

In summary, we finally included six readily available clinical indicators to predict refractory *M. pneumoniae* pneumonia. This predictive scale helps to determine whether a child will develop refractory *M. pneumoniae* pneumonia early in the disease. In the retrospective and prospective cohort, the scale has good discrimination, high sensitivity and specificity.

## Abbreviations

ALT: Alanine aminotransferase
AST: Aspartate aminotransferase
CK: Creatine kinase
HB: Hemoglobin
LDH: Lactate dehydrogenase
MPP: *M. pneumoniae* Pneumonia
RMPP: Refractory *M. pneumoniae* pneumonia
WBC: White blood cells
